# Patient Preferences in Rare Diseases: A Qualitative Study in Neuromuscular Disorders to Inform a Quantitative Preference Study

**DOI:** 10.1007/s40271-020-00482-z

**Published:** 2021-02-27

**Authors:** A. Cecilia Jimenez-Moreno, Eline van Overbeeke, Cathy Anne Pinto, Ian Smith, Jenny Sharpe, James Ormrod, Chiara Whichello, Esther W. de Bekker-Grob, Kristin Bullok, Bennett Levitan, Isabelle Huys, G. Ardine de Wit, Grainne Gorman

**Affiliations:** 1 Translational and Clinical Research Institute, Newcastle University, Newcastle-Upon-Tyne, UK; 2 Evidera, London, UK; 3 Clinical Pharmacology and Pharmacotherapy, University of Leuven, Leuven, Belgium; 4 Pharmacoepidemiology Department, Center for Observational and Real-world Evidence, Merck & Co, Inc., Rahway, NJ USA; 5 Julius Center for Health Sciences and Primary Care, University Medical Center Utrecht, Utrecht University, Utrecht, The Netherlands; 6 Muscular Dystrophy UK, London, UK; 7 School of Applied Social Science, University of Brighton, East Sussex, UK; 8 Erasmus School of Health Policy and Management, and Erasmus Choice Modelling Centre, Erasmus University, Rotterdam, The Netherlands; 9 Global Patient Safety Department, Eli Lilly & Co., Indianapolis, IN USA; 10 Janssen Research and Development, Titusville, NJ USA

## Abstract

**Introduction:**

It has become increasingly important to include patient preference information in decision-making processes for drug development. As neuromuscular disorders represent multisystem, debilitating, and progressive rare diseases with few treatment options, this study aimed to explore unmet health care needs and patient treatment preferences for two neuromuscular disorders, myotonic dystrophy type 1 (DM1) and mitochondrial myopathies (MM) to inform early stages of drug development.

**Methods:**

Fifteen semi-structured interviews and five focus group discussions (FGDs) were held with DM1 and MM adult patients and caregivers. Topics discussed included (1) reasons for study participation; (2) disease signs/symptoms and their impact on daily lives; (3) top desired benefits; and (4) acceptability of risks and tolerance levels for a hypothetical new treatment. Data were analyzed following a thematic ‘code’ approach.

**Results:**

A total of 52 participants representing a wide range of disease severities participated. ‘Muscle strength’ and ‘energy and endurance’ were the disease-related unmet needs most often mentioned. Additionally, improved ‘balance’, ‘cognition’ and ‘gut function’ were the top desired treatment benefits, while ‘damage to the liver, kidneys or eyes’ was the most concerning risk. Factors influencing their tolerance to risks related to previously having experienced the risk and differentiation between permanent and temporary risks. A few differences were elicited between patients and caregivers.

**Conclusions:**

This qualitative study provided an open forum to elicit treatment-desired benefits and acceptable risks to be established by patients themselves. These findings can inform decisions for developing new treatments and the design of clinical trials for DM1 and MM.

**Electronic supplementary material:**

The online version of this article (10.1007/s40271-020-00482-z) contains supplementary material, which is available to authorized users.

## Key Points for Decision Makers

**Table:** 

This research serves as a case study for preference studies at an early stage of drug development where there are no approved treatment options and little is known about potential treatment profiles.
For patients with myotonic dystrophy and mitochondrial disorders, ‘muscle strength’ and ‘energy and endurance (and daytime sleepiness)’ were the two symptoms for which patients would like to see improvement.
The ‘permanency (or temporality)’ of the risk was more relevant to patients when considering risk acceptability than the type of risk itself.
The majority of important treatment attributes were shared by patients with myotonic dystrophy and mitochondrial disorders, supporting the design of an elicitation experiment using a common survey.

## Introduction

Rare diseases are often characterized as complex disorders that are uncommon, and manifest as serious and debilitating conditions with often poor prognoses [1]. There are many challenges in the development of new treatments in the context of rare diseases [2]. Small patient numbers, genotypic and phenotypic heterogeneity, and lack of natural history data leave drug developers and evaluators with a degree of uncertainty when making decisions on the development of potential new therapeutic agents. To expedite the development of treatments and to advance the understanding of rare diseases, stakeholders (e.g. industry, regulators and payers, and health-technology assessment [HTA]) are increasingly seeking input from patients with rare diseases throughout the drug development lifecycle [2–5]. Patient preference information can better inform decision makers regarding the general unmet need of the patient population and the potential value of new treatment developments, relevant clinical trial endpoints, and benefit–risk decision-making processes across the product development lifecycle [6–8]. Patient preference information is uniquely important for the development of new treatments for rare diseases, as clinical trial evidence is often considerably uncertain or variable given the rare nature of the disease and the challenges affecting trial enrollment. Moreover, the patients’ voice can complement existing trial evidence used for decision-making processes [9–11]. However, collecting this type of information, particularly in rare diseases, is not always straightforward as there are no established treatments or treatment profiles to serve as a starting point for further research [12, 13].

Neuromuscular diseases (NMDs) are rare diseases recognized as primarily affecting the muscle [14, 15]. Two specific forms of NMDs are myotonic dystrophy type 1 [DM1] and mitochondrial myopathies [MM]) [14, 15], which together affect around 20 people in every 100,000 worldwide [15, 16]. These two diseases are highly heterogeneous and, in addition to muscle weakness, commonly affect other body systems, including the central nervous system. Symptoms such as learning difficulties, daytime sleepiness, fatigue and cognitive defects have been described in a majority of patients, with higher prevalence and severity in younger populations [17, 18]. Given the debilitating nature of the disease, a sizeable number of DM1 and MM patients may require caregiver support to perform daily activities and to administer personal care [19, 20]. With limited treatment options, current standards of care focus on the management of symptoms.

Patient preferences can be described as treatment attributes or features that matter most to patients, and the relative importance of those attributes or trade-offs that patients are willing to make, e.g. what degree of risk would patients tolerate to experience a certain degree of benefit [21]. A variety of methods to collect patient preferences have been identified and grouped as either methods for exploration (qualitative methods) or methods for elicitation (quantitative methods) [22]. Quantitative methods elicit the desirability or acceptability of treatment alternatives by asking patients to express their preferences for attributes (i.e. treatment features) and levels (i.e. different values that the treatment features can attain), or by requesting them to make a preferred choice between different treatment (benefit–risk) profiles [22]. However, to ensure the correct design of quantitative studies, information gathered in qualitative studies is crucial. Regulators have highlighted the importance of performing qualitative studies prior to quantitative studies [23, 24]. Initial qualitative research (e.g. implementing interviews and focus groups) can provide valuable insights regarding the disease and identification of (unmet) health care needs [9–11].

This qualitative study aimed to collect patient and caregiver insights regarding unmet health care needs (i.e. signs and symptoms of disease and their impact on daily life), desired treatment benefits, and acceptability of treatment risks in two NMDs—DM1 and MM [12]. The results of this study are intended to inform the design of a subsequent quantitative patient preference study in DM1 and MM by exploring treatment attributes, attribute levels and terminology as defined by participants themselves. This research serves as a model for future rare diseases research at an early stage of drug development where little is known about potential treatment profiles and where patient preference information may help pharmaceutical companies to define treatment target profiles.

## Methods

This protocol and associated patient consent materials were reviewed and approved by the Ethics Committee at Newcastle University, UK (ref: 8840/2018). Adherence to guidelines for Reporting Qualitative Research was undertaken for this report [24].

### Study Design

This study consisted of semi-structured interviews and focus group discussions (FGD) with DM1 and MM patients and caregivers. The study was based on the hypothetical case scenario of a putative treatment at the early stages of development. Interview and FGD guidance (see the electronic supplementary material [ESM]) were established covering four main themes: (1) reasons for participating in the qualitative study (*“Can you tell us a little bit more about yourself and why you decided to participate on this study?”*); (2) disease signs/symptoms and their impact on activities of daily living [ADL; referred to as unmet health care needs] (*“In general, can you give us an example of how any of these symptoms affects your daily life?”*); (3) desired treatment benefits and focus (*“… a new treatment could be available for you tomorrow, which symptom would you like to be cured from first? With what type of improvement would you be satisfied?”*); and (4) acceptability of risks (and those feared the most) from the list of potential risks (*“… how do you feel about these? Is there any of these that you fear the most?”*) and potential risk tolerance (*“If there could be a treatment attempting to improve the worst of your disease symptoms, is there any of these that you would or would not tolerate?”*) [see the ESM].

### Patient Engagement and Support

Given the importance of engaging patients as research partners, the design of this study has been informed by four patient representatives (two patients with DM1 and two caregivers of DM1 and MM patients), who have contributed to the design and interpretation of results. The patient representatives have regularly participated in weekly team meetings, revised study materials, and one patient representative has validated the interpretation of results as an independent coder of the study transcripts. Their support and contributions to the study design have ensured the delivery of a truly patient-centered study design with greater generalizability to a wider group of participants.

### Literature Review

To support the development of the discussion guidelines and to generate a list of potentially relevant treatment attributes, recent publications regarding symptom prevalence in DM1 and MM were reviewed [25–27]. The list of potential risks was established through a review of adverse events reported in publications on past and current clinical trials in these diseases, retrieved through a literature search via PubMed Central^®^ and ClinicalTrials.gov (search completed between December 2018 and February 2019).

### Sample

The goal was to recruit a minimum of ten participants in face-to-face (one-on-one) interviews and four FGDs, each consisting of six to eight participants, to achieve data saturation (i.e. point at which the chance of obtaining new information with additional participants was considered minimal) [28, 29]. Participants were recruited via the UK Myotonic Dystrophy Patient Registry and allied patient organizations: Muscular Dystrophy UK (MDUK), Cure Congenital Myotonic Dystrophy (Cure DM CIC), the Myotonic Dystrophy Support Group (MDSG) and Lily Foundation (mitochondrial myopathy). Recruitment strategies were organization-dependent. The UK DM Patient Registry advertised the study via direct email to their registrants, whereas promotion for the other organizations was via social network platforms and/or via ‘patient days’ where FGDs and interviews would form part of the event.

Sample selection criteria were chosen to reflect the expected sample to be recruited for future quantitative studies. All participants were aged ≥ 18 years and were independently able to provide informed consent. Self-reported DM1 and MM patients were classified into two groups, defined by age at onset of symptoms to reflect expected differences in cognitive abilities: Group 1, patients with early disease onset (first experienced symptoms reported before 20 years of age); and Group 2, patients with late disease onset (first experienced symptoms on or after 20 years of age). A third group was made up of caregivers who were defined as spouse, partner, parent(s), legal guardian or other adult close to the family, either living in the same house or in contact with the patient in a caregiver role, providing support at least four times/week for at least 1 h or more, for the last 12 months or longer. The aim of the study was not to understand caregivers’ preferences but to provide an insight into what they judged to be important for the patients they cared for, which may otherwise go unrecognized by patients themselves due to the nature of their disease [30]. Participants of Groups 2 and 3 were invited for FGDs, while participants allocated to Group 1 were invited for face-to-face interviews to allow for a more direct discussion and avoid overwhelming them in a group setting; however, participants could change the interview/discussion method if deemed necessary based on their preference and availability.

### Data Collection

Face-to-face interviews were conducted either online or in person and were primarily offered to patients belonging to Group 1. Interviews were carried out as semi-structured interviews, lasting about 45 min and following the predefined guidelines. FGDs were held at three different geographical locations across the UK: London, Nottingham and Newcastle upon Tyne. They were conducted at neutral venues outside of clinical premises and led by one moderator (a clinical research associate experienced in these two disease groups) who followed the predefined guidelines, with a presentation of a set of slides to facilitate the discussion.

Participants provided basic demographic information and completed a tool to measure patient-reported levels of functional impairment (i.e. the DM1-Activ^C^), which is a 25-item Rasch-developed questionnaire [31], with scores ranging from 0 to 50, where the higher the score, the more capable the respondent reports to be when performing common tasks of daily living. A second patient-reported outcome was completed by caregivers only, to assess caregiving-associated stress levels, i.e. Caregivers Strain Index (CSI) [32], which is a 12-item questionnaire where a score of 7 or higher would indicate high stress levels.

Additionally, all participants were requested to rank their top five priority attributes from the predefined treatment-attributes list (*n *= 15) generated from the literature review [25, 33]. Participants were permitted to add as many attributes to the list from their own experience as desired (see the ESM).

### Data Management

All participants provided informed consent prior to study participation. Interviews and FGDs were audio-recorded, transcribed *verbatim*, pseudonymized (i.e. removing identifiable data to ensure privacy and confidentiality), and then securely stored at Newcastle University, UK.

### Data Analysis

Data obtained from interviews and FGDs were analyzed simultaneously, and transcripts were analyzed through framework analysis [34, 35]. The data were interpreted for patterns, consensus, and critical observations across participants, which resulted in the formulation of thematic ‘codes’. An initial thematic framework (or coding tree) was created matching the main topics in the interview guides. Then, during a ‘familiarization process’, researchers became aware of key themes through the conduct of the interviews and FGDs, and, by reading the transcripts, confirmed the applicability of the initial coding tree. Four researchers independently examined one FGD transcript in detail to construct over the initial thematic framework (coding tree) and add subcodes as agreed [36]. Subsequently, one researcher coded all FGDs and interview transcripts using the final agreed framework. Verbatim sections of transcripts that corresponded to a theme were selected and placed in the predefined framework where they were linked to a corresponding theme. These themes were then analyzed and interpreted for agreement between responses from the different subgroups (i.e. groups 1–3). During the process of analysis, the branches of the coding tree changed slightly as additional inductive codes were included, but the main codes remained as initially defined (see the ESM). The coding process was completed using Microsoft Office Excel (for Mac 2011, version 14.7.7).

Sociodemographic and clinical data were summarized using descriptive statistics (e.g. mean, standard deviation, minimum–maximum and frequency). Disease groups were compared using the independent group *t*-test or Chi-square test.

## Results

### Study Sample

A successful recruitment rate resulted in a total of 52 participants (33 patients and 19 caregivers) volunteering for the study, completing a total of 15 interviews (two of which were performed remotely and two of which were dyadic interviews including both caregivers of the same patient as one) and five FGDs with between five and nine participants each (Table 1). Most participants (75%) represented DM1. Five caregivers (DM1) and two patients (DM1) with late disease onset participated in face-to-face interviews instead of FGDs, given their lack of availability for FGD dates and locations. In contrast to expectations, the two MM patients with early disease onset chose to attend FGDs over face-to-face interviews because of the opportunity to interact with other patients and the opportunity for their family members to attend. This reallocation to the FGD was considered appropriate based on the clinical judgment of the researchers. Five caregivers represented pediatric patients (< 18 years of age). Compared with MM caregivers, DM1 caregivers represented older patients. Only 20% of patients self-reported as full-time employees, in contrast to the caregivers’ group (58%).

**Table 1:** Patient and caregiver demographics

	DM1	MM	All
Patients			
*n*	24	9	33
Age, years [median (SD, range)]	47.3 (11.51, 33–72)	45.8 (17.44, 19–75)	46.9 (12.94, 19–75)
Age of first symptom, years [median (SD, range)]	23.7 (14.67, 0–60)	29.6 (15.99, 5–53)	25.2 (115.0, 0–60)
First symptoms age <20 years (%)	9 (37.5)	2 (22.2)	11 (33.3)
Years since first symptom [median (SD, range)]	23.6 (12.43, 5–46)	16.0 (11.64, 0–35)	21.7 (12.50, 0–46)
Taking medication for disease-specific issues [*n* (%)]			
Yes, currently	10 (41.7)	6 (66.7)	16 (50)
Yes, in the past	2 (8.3)	0	2 (6.3)
No, never	12 (50.0)	2 (22.2)	14 (43.8)
No, never taken or considered at all	8 (33.3)	2 (22.2)	10 (31.3)
No, never taken but discussed with doctor or heard about from a friend	4 (16.6)	0	4 (12.6)
Employment status [*n* (%)]			
Yes, full-time	5 (20.8)	2 (22.2)	7 (21.2)
Yes, part-time	8 (33.3)	0	8 (24.2)
No employment	9 (37.5)	4 (44.4)	13 (39.4)
Student	0 (0.0)	1 (11.1)	1 (3.0)
Retired	2 (8.3)	1 (11.1)	3 (9.1)
Other	0 (0.0)	1 (11.1)	1 (3.0)
DM1-Activ-C score [median (SD, range)]	35.3 (12.80, 8–50)	16.0 (12.49, 10–46)^a^	32.2 (13.58, 8–50)
Caregivers			
*n*	14	5	19
Age, years [medium (SD, range)]	58.0 (12.76, 42–76)	45.2 (8.96, 38–55)	54.6 (13.00, 38–76)
Age of person they care for, years [median (SD, range)]	34.9 (15.13, 4–66)	10.7 (14.15, 2–27)^a^	30.4 (17.4, 2–66)
Employment status [*n* (%)]			
Yes, full-time	6 (42.9)	5 (100.0)	11 (57.9)
Part-time	2 (14.3)	0	2 (10.5)
No employment	4 (28.6)	0	4 (21.1)
Student	0	0	0 (0.0)
Retired	1 (7.1)	0	1 (5.3)
Other full-time caregiver	1 (7.1)	0	1 (5.3)
DM1-Activ-C score [medium (SD, range)]—for patients ≥ 16 years of age only	26.2 (16.22, 0–49)	12.8 (10.94, 2–28)	25.8 (17.2, 0–49)
Caregiver Strain Index Rating			
Score [medium (SD, range)]	6.7 (3.87, 1–13)	9.0 (1.58, 7–11)	7.3 (3.53, 1–13)
Categories [*n* (%)]			
No additional stress	0 (0.0)	0 (0.0)	0 (0.0)
Some additional stress, possible need for intervention	7 (53.8)	0 (0.0)	7 (38.9)
High level of stress, i.e. score ≥ 7	6 (46.2)	5 (100.0)	11 (61.1)

*SD* standard deviation, *DM1* myotonic dystrophy type 1, *MM* mitochondrial myopathy

^a^Significant difference between groups *p* < 0.05

DM1 patients had higher DM1-Activ-C scores (i.e. less impaired) than MM patients (35.3 ± 12.8 vs. 16.0 ± 12.5, *p* = 0.03). Similar results were observed in the caregiver groups, however the differences were not significant. While there was no significant difference in the mean CIS score between groups of caregivers, all of the MM caregivers (100%; *n *= 5) reported a ‘high level of stress (≥ 7, indicating a high level of stress [32]) versus 46% (*n *= 14) for DM1. The item that both disease groups reported consistently as a stress factor was ‘changes in personal plans, e.g. had to turn down a job; could not go on vacation)’.

### Reasons for Participating

Reasons for participating in either face-to-face interviews or FGDs were grouped into three categories (a–c). The most common reason (49%) was (a) altruistic behaviors (i.e. contributing to the welfare of others). The chance of helping others was a common encouragement, *“… help somebody else …”* (patient), either family members or future generations; *“My daughter has [the disease] and that is why I am doing this, anything that I can do that eventually will help her …” *(caregiver). Another frequently mentioned altruistic reason was purely to support research; *“basically I think any information that you can get out there could help”* (patient). Besides these altruistic reasons, the other reasons for study participation were due to (b) personal interest in the study (27%), i.e.* “I’m doing this because it sounds interesting”* (patient), *“I’m interested to know more about what's going on with research”* (caregiver), and/or (c) a belief in how this study could help lead to a cure (24%), *“I think anything that could lead to a treatment or medication is excellent …”* (patient).

### Unmet Health Care Needs

The theme of unmet health care needs was divided into two main codes: (1) disease signs and symptoms, and (2) impact on ADLs. The most frequently mentioned issues related to ‘muscle strength’ (33%) and ‘energy and endurance’ (29%), which included fatigue, daytime sleepiness and exercise intolerance (Table 2). Balance, cognition, speech, and gut- and cardiovascular-related issues were also frequently reported by patients. Participants, both patients and caregivers (*n *= 18), would refer to other family members affected by the disease as comparators to explain different disease scenarios (either milder or more severe) *“cause what my brother have I don’t, he's got a worse disease than I am, and he's really, debilitated”*. Unmet health care needs were often disease-specific, with ‘daytime sleepiness’ or ‘myotonia’ only reported by DM1 patients or their caregivers, while issues such as organ failure and hearing problems were exclusive to the MM group. Cognition, swallowing and respiratory issues were mentioned almost twice as much by caregivers than by patients, in both disease cohorts (see the ESM).

**Table 2:** Top unmet health care needs as elicited from DM1 and MM patients and caregivers

Code	*C* [*N*(*P*)]	MB	Symptom examples	Impact on: (*n*)			ADL examples
				Work	Social and leisure	ADL	
Muscle strength	69 [*n *= 34, *n *= 29 (20P) DM1; and *n *= 5 (3P) MM]	12	*“The weakness in my hands”* *“I have … foot drop?”* *“And his strength on his hands …”*	6	19	52	*“Like peeling potatoes … I would said. I don't cut stuff like that cause I got no pressure (on hands) …”* *“I fell over, cause [uhm] I couldn’t lift my feet”*
Energy, endurance and daytime sleepiness	56 [*n *= 21, *n *= 16 (13P) DM1; and *n *= 5 (3P) MM]	4	*“I am sleepy all the time”* “He falls asleep just like that”	5	9	43	*“When I am sleepy it affects my physical abilities”* *“He could sleep forever … I would struggle to wake him up”*
Cognition and learning difficulties	23 [*n *= 16, *n *= 12 (5P) DM1; and *n *= 4 (3P) MM]	4	*“I can’t think quickly”* *“I mean his memory …”* *“Concentration is not there”*	11	11	18	*“I could be sitting in the meeting and suddenly clicks in and that's it[!] and everybody could be talking Chinese for all I know”*
Balance and coordination	21 [*n *= 15, *n *= 11 (10P) DM1; and *n *= 4 (3P) MM]	1	*“I am in average falling every ten days”* *“The difficulties in balance”*	5	15	18	*“He can't play golf … he can't stand and hit a ball”* *“He was a builder and he fell 3 stories”*
Cardiovascular fitness	21 [*n *= 13, *n *= 11 (8P) DM; and 2 (1P) MM]	0	“I am obviously concerned about my heart” “I have a pacemaker”	0	3	4	*“His blood pressure changes with heights quite drastically like in altitudes when going skiing”*
Speech	18 [*n *= 15, *n *= 11 (4P) DM1; and *n *= 4 (1P) MM]	3	“Speech as well … it tends to get slurred” “Suffers with dysarthria”	0	17	18	*“His speech is indistinct and when he gets excited you ... you can't follow”*
Gut	18 (*n *= 15, *n* = 13 (9P) DM1; and *n *= 2 (1P) MM]	3	*“Toilet problems”* *“What affects me the most is my stomach issues”*	0	3	13	*“I got diarrhea and constipation from 1 day to the other”* *“Well … he still uses nappies …”*
Mood and motivation^a^	15 [*n *= 9, (2P) DM]	3	*“I have apathy”* *“Depression”* *“It was the lack of motivation”*	0	1	8	*“I cannot get motivated to do anything”*
Swallowing	14 [*n *= 9, *n *= 8 (4P) DM1; and *n *= 1 MM]	1	*“Gagging when eating”* *“Had to stop to swallow saliva”* *“He is PEG fed”*	0	4	8	*“My husband ... stops in his conversation and consciously swallow cause its ... his saliva builds up”*
Myotonia^a^	14 [*n *= 10 (8P) DM]	0	*“A tightness in my muscles”* *“It is difficult to let the hand go”*	0	3	11	*“To have to ask a stranger on the tube, to open a bottle of water for me”*
Respiratory system	12 [*n *= 7, *n *= 5 (2P) DM1; and *n *= 2 MM]	0	*“Respiratory failure”*	0	1	5	*“He is on BiPap overnight”*

*C* number of times the code was mentioned, *N* number of participants mentioning each code, *P* number of those participants who were patients, *MB* number of times the code was highlighted as the sign or symptom that was most bothersome to the patient, *DM1* myotonic dystrophy type 1, *MM* mitochondrial myopathies, *ADL* activities of daily living, *PEG* percutaneous endoscopic gastrostomy

^a^ Codes exclusively identified in the DM group

Other signs and symptoms that were discussed included pain, vision, hearing, hand dexterity (exclusive to DM1), seizures and organ failure (exclusive to MM) [not shown in Table 2]. Previous experiences associated with the progression of the disease, and fears regarding the uncertainty of future progression, were topics that worried patients and their caregivers among both disease groups; *“the progression, and knowing that things are not necessarily going to get any better but they are going to get worse”* (patient); *“because you don't know how things are going to be for him …” *(caregiver).

Some of these signs and symptoms interfered with social/leisure and/or work/school/housework activities (Table 2). Signs and symptoms that impacted ADL related to social/leisure activities the most included ‘speech’ and ‘balance and coordination’. There were 37 examples relating to how the disease affected or impaired performance in work-related activities; however, they did not point to one sign or symptom in particular but rather to the disease in general (see the ESM).

While discussing unmet health needs, health care interventions (e.g. cataracts surgery, hearing aids, percutaneous endoscopic gastrostomy [PEG], speech therapy and orthotics) were mentioned, together with some lifestyle changes such as diet, vitamin supplements or energy drinks, and physical activity. Only one medication, which is recommended for daytime sleepiness, was mentioned during one of the DM1 FGDs and during one face-to-face interview.

**Fig. 1:**
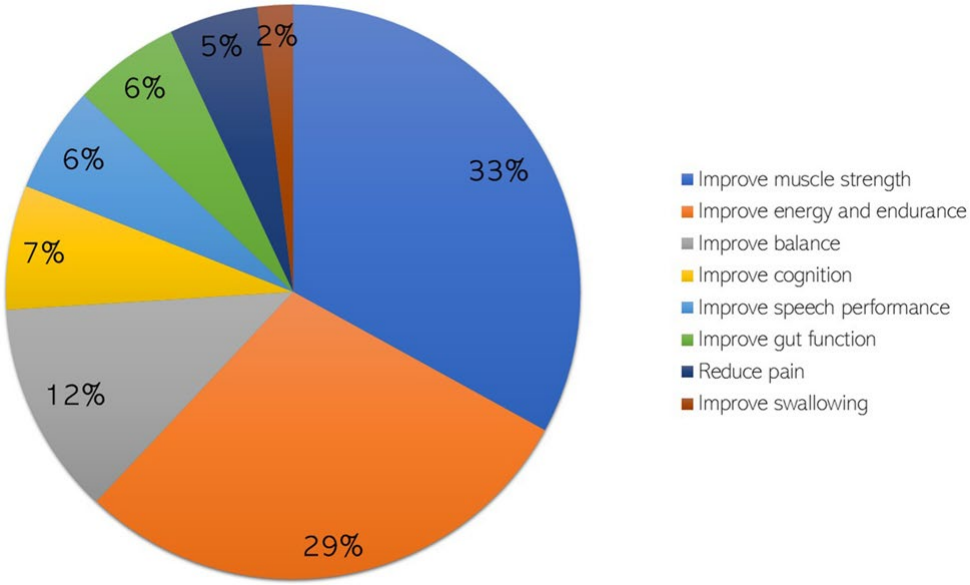
Most-desired benefits as expressed by DM1 and MM patients and caregivers during focus groups and interview discussions. This figure tallies the results obtained when discussing topic 1 (unmet health care needs), topic 2 (desired treatment benefits), and from the ranking list count. *DM1* myotonic dystrophy type 1, *MM* mitochondrial myopathies

When combining responses obtained to the open-ended questions about signs and symptoms (i.e. unmet health care needs), to issues from which the patient would like to be cured first and those they would like to improve if a complete cure was not possible, the two top attributes (i.e. improving ‘muscle strength’ and ‘energy and endurance’) were consistent across both disease groups (Fig. 1).

### Desired Benefits

During this discussion, participants were able to describe desirable treatment attributes, and provided examples of what a satisfactory improvement in signs and symptoms would mean. For example, when mentioning minimum expected improvements related to ‘muscle strength’, participants mentioned they would want at least to be able to *“continue to cycle …”* (patient) or *“go from sitting to standing with no problem …”* (patient), or *“play golf again…”* (patient and caregiver).

Differences identified between the ranking list scores and the list obtained from the qualitative data (i.e. Table 2) demonstrated the following: (1) in the ranking list, ‘improve energy and endurance’ scored higher than ‘improve muscle strength’, whereas during the discussion, ‘muscle strength’-related symptoms were mentioned more often than those related to ‘energy and endurance’; (2) in the ranking list, ‘improve gut function’ ranked in the top 5, whereas when counting the times it was mentioned during the discussions, this symptom would be listed lower in ranking (position 7 in Table 2); (3) the option ‘slow down disease progression’ ranked third in the ranking list, whereas this term was not included as a code when analyzing the qualitative data, as the progression of the disease was always mentioned linked to a specific symptom.

The ranking exercise allowed us to elicit desired benefits not previously included in the list, such as improve lack of motivation, improve motor function, improve coordination, improve facial expression, improve sleep at night, improve organization/memory skills and reduce seizures. When applicable, these additional desired benefits would be grouped and counted as they would have following the same criteria used when analyzing the qualitative data; for example, improved facial expression was counted as ‘improve muscle strength’ and organization/memory skills would have been counted as ‘improve cognition’. Some of these (e.g. improve sleep at night and reduce seizures) are disease-specific symptoms; these were then excluded when deciding which attributes to bring forward to the quantitative study.

### Acceptability of Risks

To discuss risks, participants were initially presented with a list of potential risks (see the ESM) and were asked to choose and rank the risks they were most concerned about in order of importance (Table 3). There were no significant differences between disease groups on the prevalence of patients reporting a risk feared or a risk to be tolerated. ‘Damage to the liver, kidneys or eyes’ was feared by most participants (42%; *n *= 16 DM1, and *n *= 5 MM). Risks most often categorized as ‘tolerated’ were ‘headaches or pain in the muscle or bone’ (40%; *n *= 7 DM1, and *n *= 1 MM) and ‘emotional or behavioral effects’. A few participants (DM1) added ‘other’ risks, not listed, such as vomiting, mental illness or death.

**Table 3:** Ranking of risks and percentages of participants who quoted the risk

Rank	Type of risks mentioned as ‘feared the most’	Percentage of participants (caregiver percentage)
1	Damage to the liver, kidneys or eyes	42 (26)
2	Emotional or behavioral effects such as insomnia, anxiety, depression and irritability	20 (36)
3	Heart-related effects such as chest pain or palpitations	15 (50)
4	All of them	7 (50)
5	Stomach or gut effects such as nausea, lack of appetite and diarrhea	5 (67)
6	Other (i.e. as elicited directly from the participant)	5 (25)
7	Headaches or pain in the muscle or bone	4 (0)
Tolerated risks in the return of potential benefits		
1	Headaches or pain in the muscle or bone	40 (40)
2	Stomach or gut effects such as nausea, lack of appetite and diarrhea	30 (40)
3	Heart-related effects such as chest pain or palpitations	10 (33)
4	Emotional or behavioral effects such as insomnia, anxiety, depression and irritability	10 (25)
5	Damage to the liver, kidneys or eyes	5 (50)
6	All of them	5 (0)

The vast majority of participants [*n *= 39; *n *= 34 (29 participants were patients) DM1; and *n *= 5 (5 participants were patients) MM] mentioned that some of these risks are similar to symptoms of their diseases, and provided examples of how these affect them in daily life as well as coping mechanisms or treatment strategies to tackle them. Some described desirable treatment attributes, and participants (25%) expressed how the level of tolerance for specific risks was related to previous times experiencing that same risk, either as an adverse event of a medication or as manifestation of the disease itself. For example, one patient (DM1) with a pacemaker indicated that he would accept the risk of ‘heart-related effects’ in exchange for improvements in ‘balance and coordination’ because the patient felt safer having a pacemaker. Another participant with the same NMD (i.e. DM1) found ‘heart-related effects’ to be a non-tolerated risk, as his heart was susceptible for complications and he was not eligible to receive preventive treatment (such as a pacemaker). Another patient mentioned that ‘stomach or gut effects’ are tolerable as he/she *“already got gut problems and diarrhea, so I can live with that …”*.

Among the most feared risks, ‘damage to the eyes’ was specifically mentioned as the most relevant of the three organs (i.e. eyes, liver and kidney), and the importance of certain words and their impact on risk perception was observed. Emphasis on the importance of the word ‘damage’ was noted, as ‘damage to the eyes’ was perceived as permanent and seen as a higher risk level (e.g. feared most or not tolerated) when compared with temporary damage in relation to liver, kidneys or eyes.

## Discussion

This study explored the unmet health care needs and treatment expectations of patients (as mentioned by patients and caregivers) with two different types of neuromuscular disorders (DM1 and MM). We identified the most frequently reported signs and symptoms that these two diseases have in common and that appear to most impact patients’ ADLs. We identified which of these signs and symptoms patients would prioritize if a treatment could become available, and obtained initial insights regarding the acceptability of risks in exchange for treatment benefits. This study was performed with no treatment profile in mind as we aimed to inform any potential stakeholder with assets at the early stages of the drug development lifecycle that could benefit these groups.

Given the rare nature of these diseases, and generally similar clinical manifestations, we aimed to provide insights that could inform a quantitative study targeting any of these NMDs. The DM1-ActivC scores identified scores in this DM1 patient sample that were significantly lower than those reported in populations participating in clinical trials that usually demand a higher level of functional capacity [37, 38]. These two patient cohorts appear to suffer from similar unmet health care needs, and combining their results may benefit from a larger sample for the quantitative study [12]. Patients affected by these two diseases have little or no experience of medications, and health care experiences vary from patient to patient, even within the same health care service model (UK). Interestingly, when discussing reasons for participating in this study, there was a commonly expressed belief that this study could lead to the development of a new treatment, while recognizing this may not available within their own lifetime.

The combination of different qualitative methodologies (i.e. interviews and FGDs) and approaches (face-to-face and remote interactions) allowed widened participation, with the involvement of often hard-to-reach participants living in remote areas, with speech disturbances, or with limited walking capabilities. In these disease groups, both patients and caregivers are usually involved in, and affected by, treatment decisions [11]. The inclusion of caregivers in this study allowed (1) representation of pediatric patients, and (2) insights into unmet health care needs that, although relevant, patients may not mention themselves as much as caregivers, such as swallowing and respiratory complications, both of which would be deemed as severe manifestations with potential impact on survival [39, 40]. In the study of van Overbeeke et al. [23], DM1 patients and HTA representatives suggested to explore caregiver preferences, ‘instead’ of patient preferences, in specific cases (such as DM1) where patients may have impaired cognitive function and where low disease acceptance or disease insights by patients may create a wrong perception of the issue. This could explain why more DM1 caregivers (*n *= 7/14) reported cognitive issues than patients (*n *= 5/24) [Chi-square *p *< 0.05]. Another explanation could be that some of these caregivers represented more affected, vulnerable patients whose disease burden precluded participation. An issue that exemplifies this difference was the use of a PEG tube (feeding tube), as none of the patients reported the use of a PEG tube but several caregivers did. These results cannot fully support the argument of the inclusion of caregivers ‘instead’ of patients, but we can argue that their inclusion provides additional information to understand preferences that may otherwise remain unheard.

Although counts of symptom prevalence in DM1 and MM have been described before, either from anecdotal evidence from health care specialists or from surveys among patients [25, 27, 33], this study provides additional direct evidence of what is most important to patients, and allowed patients to directly express their opinions in their own words. Examples of terminology used by patients include the way they referred to myotonia more commonly as ‘stiffness’, or described cognitive issues as ‘lack of concentration’ or ‘different learning needs’. This study has allowed us to explore why these symptoms (or disease-related issues) are so important to patients and to obtain examples of how they affect ADLs, examples that may be used when introducing an attribute as part of a quantitative survey or that can inform the selection of outcome measures when designing a clinical study.

For future (quantitative) patient preference studies, the frequency with which signs and symptoms were mentioned, and the wording used by participants in this study, can be considered when defining treatment attributes. In this study, the top two unmet health needs (i.e. ‘muscle strength’ and ‘energy and endurance’) are in line with top symptoms previously reported for both of these diseases [25, 33]. These two unmet health needs were consistently mentioned as most important across the different discussion topics, i.e. as top unmet health care needs, top desired benefits and top ranked attributes in the ranking exercise, and therefore we would consider these two unmet needs as potential attributes of treatment benefits that could be included in a quantitative study.

The examples given for ‘impact on ADLs’ and the ‘minimum level of expected improvement’ can guide stakeholders in the inclusion of patient-relevant endpoints in clinical trials. Some functional outcome measures currently explored in clinical trials in both disease cohorts relate to needs observed in the current study. For example, outcomes such as the Timed Up and Go or Sit-to-Stand tests relate to the expressed need of *“to be able to go from sitting to standing with no problem …”*. However, some other needs remain poorly investigated in clinical settings, such as ‘gut’, ‘cognition’ and ‘speech’ issues or needs associated with upper limb strength and functionality. The impact of ‘speech’ and ‘balance and coordination’ on ADLs was highlighted and the development and implementation of strategies to reduce the level of their interference should be encouraged. For example, talking on the phone was one ADL example mentioned repetitively when discussing ‘speech’ issues, and, more than once, auxiliary visual aids were mentioned as strategies to support phone communication. This information (i.e. impact on ADLs) could inform outcome measures in future clinical trials.

The ranking exercise allowed us to obtain a first glimpse into the value patients attached to each potential treatment benefit when requested to choose only the top five and rank them in order of importance. Interestingly, when given this choice, ‘improve energy and endurance’ ranked higher than ‘improve muscle strength’, and this was consistent in both disease groups. In the ranking exercise, ‘slow down disease progression’ was ranked in third place, however this was never an isolated issue when discussed openly, where it would always be referred to as the progression of a symptom and therefore coded as such. When translating this into a quantitative study, we are aware of the fact that these diseases do not progress in a standard manner and each symptom can differ in progression speed and can vary from patient to patient; therefore, we would not advise to use ‘overall disease progression’ as such. For our study, we decided to include progression, only in connection to specific symptoms but not as an independent attribute.

The ‘risks feared the most’ were ranked in inverse order of the risks mentioned as tolerated, which validated the consistency of these results. Interestingly, when risks were discussed, participants identified some of them as actual symptoms of the diseases. Having experienced any of these risks as actual disease symptoms was a clear factor influencing the perception of the risk, either by reducing the fear towards the risk or by making participants more sensitive to it. Interestingly, the word ‘damage’ and the feeling of losing control over the risk were also found to be important factors influencing patients’ trade-offs. We identified that regardless of the risk chosen, it was important to highlight the permanency or temporality of the effect as a way to define the severity of the risk or the possibility of patients having ‘control’ over an adverse event that, with time, could be recovered.

### Strengths and Limitations

The design of the interview and the FGD guides was informed by a literature review and discussion with health care professionals as well as patients and caregivers. This approach may differ from other qualitative patient preference studies in which the codes and treatment attributes are predefined and the aim is to build understanding about patients’ attitudes towards these. This study design allowed patients and caregivers to first openly express their needs and expectations (bottom-up), before any predefined options were presented to them (top-down). Interviews and focus groups were always moderated by the same researcher (CJM) to reduce variability between interviews and focus groups. The analysis was conducted by the researcher most familiar with the content of the interviews and the focus groups (CJM) and three others (EvO, IH, JO) to validate identification of themes and correct coding of the transcripts.

By nature, interviews and focus groups provide subjective evidence that may not be generalizable to different diseases, or sometimes not even to different disease spectrums. Our study ensured the inclusion of patients and caregivers from two diseases and representing a large range of severities across the disease spectrum. In the study, we showed that patients’ needs, expectations and risk tolerances are similar in the two disease populations. In this study, there was a larger sample representing the DM1 population, which not only reflects the general prevalence of this disease [41] but also the impact of recruitment attributable to patient registries (i.e. the UK DM Registry). Nonetheless, allowing recruitment via patient organizations and a patient registry permitted the inclusion of patients and caregivers with different levels of involvement in health care. Based on the DM1-Activ-C scores and the high stress scores on the CSI rates, the MM group represented a more severely affected group; however, this is difficult to compare as, to our knowledge, this is the first study using these two reported outcomes to describe an MM sample and therefore we cannot argue this is a reported outcome valid for comparison. Finally, this study only included UK participants, therefore results may not be generalizable to other health care systems where current care models for these two diseases may be different.

## Conclusion

Defining treatment attributes for preference elicitation studies is not a straightforward task when there is no treatment profile in mind. In this study, by following a qualitative design, we were able to identify (unmet) health care needs, treatment expectations, and adverse events that are feared the most, and others that may be tolerated by patients with neuromuscular disorders. ‘Muscle strength’ and ‘energy and endurance’ were the most frequently reported disease issues affecting these participants, among many relevant unmet needs that participants reported in this study that impacted their daily life. The findings of this study hold potential to inform decisions for new treatment development and the design of clinical trials for both DM1 and MM.

## Electronic supplementary material

Below is the link to the electronic supplementary material.

Supplementary file1 (DOCX 146 kb)

Supplementary file2 (DOCX 125 kb)

Supplementary file3 (DOCX 65 kb)

Supplementary file4 (DOCX 100 kb)

## Declarations

### Data Availability Statement

Material supporting the delivery of this study (i.e. interview guides and predefined ranking list) are included in this published article (and its supplementary information files). The datasets (i.e. transcripts) generated and/or analyzed during the current study are available from the corresponding author on reasonable request.
