# Correction to: Patient Preferences in Rare Diseases: A Qualitative Study in Neuromuscular Disorders to Inform a Quantitative Preference Study

**DOI:** 10.1007/s40271-021-00523-1

**Published:** 2021-05-11

**Authors:** A. Cecilia Jimenez-Moreno, Eline van Overbeeke, Cathy Anne Pinto, Ian Smith, Jenny Sharpe, James Ormrod, Chiara Whichello, Esther W. de Bekker-Grob, Kristin Bullok, Bennett Levitan, Isabelle Huys, G. Ardine de Wit, Grainne Gorman

**Affiliations:** 1 Translational and Clinical Research Institute, Newcastle University, Newcastle-Upon-Tyne, UK; 2 Evidera, London, UK; 3 Clinical Pharmacology and Pharmacotherapy, University of Leuven, Leuven, Belgium; 4 Pharmacoepidemiology Department, Center for Observational and Real-world Evidence, Merck & Co, Inc., Rahway, NJ USA; 5 Julius Center for Health Sciences and Primary Care, University Medical Center Utrecht, Utrecht University, Utrecht, The Netherlands; 6 Muscular Dystrophy UK, London, UK; 7 School of Applied Social Science, University of Brighton, East Sussex, UK; 8 Erasmus School of Health Policy and Management, and Erasmus Choice Modelling Centre, Erasmus University, Rotterdam, The Netherlands; 9 Global Patient Safety Department, Eli Lilly & Co., Indianapolis, IN USA; 10 Janssen Research and Development, Titusville, NJ USA

## Correction to: The Patient - Patient-Centered Outcomes Research 10.1007/s40271-020-00482-z

The article Patient Preferences in Rare Diseases: A Qualitative Study in Neuromuscular Disorders to Inform a Quantitative Preference Study, written by A. Cecilia Jimenez-Moreno, Eline van Overbeeke, Cathy Anne Pinto4, Ian Smith, Jenny Sharpe, James Ormrod, Chiara Whichello, Esther W. de Bekker-Grob, Kristin Bullok, Bennett Levitan, Isabelle Huys, G. Ardine de Wit and Grainne Gorman was published under the incorrect Creative Commons (CC) license (CC-BY-NC). The correct license is CC-BY. Open access for this paper was funded by Wellcome Trust, Inc. This article is licensed under a Creative Commons Attribution 4.0 International License, which permits use, sharing, adaptation, distribution and reproduction in any medium or format, as long as you give appropriate credit to the original author(s) and the source, provide a link to the Creative Commons license, and indicate if changes were made. The images or other third-party material in this article are included in the article’s Creative Commons license, unless indicated otherwise in a credit line to the material. If material is not included in the article’s Creative Commons license and your intended use is not permitted by statutory regulation or exceeds the permitted use, you will need to obtain permission directly from the copyright holder. To view a copy of this license, visit https://creativecommons.org/licenses/by/4.0/. The original article has been corrected.

