# Supplementary material for: Patient Preferences in Rare Diseases: A Qualitative Study in Neuromuscular Disorders to Inform a Quantitative Preference Study
Source: Patient. 2021 Feb 27;14(5):601–12. doi: 10.1007/s40271-020-00482-z (PMC8357717; doi:10.1007/s40271-020-00482-z)
Supplement: Supplementary file 1 — Supplementary file1 (DOCX 146 kb) [file 40271_2020_482_MOESM1_ESM.docx]

Supplementary Material 1: FGD Guidelines

BLUE = instructions to the interviewer/moderator (not to be spoken out loud)

**BOLD = eye-catcher to interviewer (important terms)**

*ITALICS = structured answer options*

Introduction

1. Before interview/focus group: read briefing on how to conduct interviews/focus groups on Project Place
2. Present yourself: say your name and that you are a researcher team member of the neuromuscular disorders case study team

Hello _________, my name is ________ (and this is (are) my colleague(s) _________) and I (we) am (are) a researcher of the PREFER team. The PREFER research project looks at how and when patient preferences for new treatments should be incorporated into the drug development process.

As described in the information sheet you previously received, you have been invited to speak with us today as someone who can provide us a good insight into myotonic dystrophy or mitochondrial disorder patient’s opinions, perspectives or preferences for potential treatments for your/their disease. This interview/focus group does not focus on assessing your responses as right or wrong but as pure source of information. We are trying to learn more about health issues that are priority for you or the patient that you care for and his or her expectations for possible treatments. However, keep in mind that this would not result in any changes to your current standards of care or medications intake from our behalf. The findings from these discussions will inform the development of an electronic survey that later on will be applied to a larger population of myotonic dystrophy and mitochondrial disorder patients and caregivers.

1. If consent has not been provided yet, proceed to consent now
2. **Demographic CRF** and **DM1-ActivC** questionnaire can be provided now
3. Thank for participation in advance at put the interview at ease of the protocol expectancies

Now, because you voluntary agreed to participate we can proceed with the interview/discussion. To facilitate our note-taking, we would like to audio record our conversations today. For your information, only researchers on the project will have access to the recordings. These recordings will be eventually destroyed after transcribed into a computer and anonymised.

We have planned this chat to last no longer than two hour(s). During this time, we have several questions that we would like to cover. We have an intermediate break schedule for the focus group discussion but if you feel the need to leave the room or the need of a break please do, as this would not affect the discussion.

1. Put group at ease: **(FGD slide 1)**

• The overall discussion will be around 1.5 to 2 hours

• You can stop the interview at any time, without having to give a reason

• You not have to answer anything they do not feel comfortable answering

• There are no right or wrong answers

• We are interested in ALL opinions

1. Discussion session starts

Now, if everyone is happy I will start the recorders *[start recording on 2 devices]*

- 1. Can you tell us **a little bit** more about yourself and why you decided to participate on this study?
- For caregivers ONLY: What is your **current role** as caregiver?
  1. Unmet health priorities: **(FGD slide 2)**

For patients: It will probably how and how much each one of you get affected by your own specific disease but from what is known about them, you may experience symptoms such as fatigue, muscle weakness, pain, cognitive impairment, gut problems, behavioural and mood changes. So now let’s talk a little bit more about your disease and how this affects your daily life.

- Can you briefly tell us about your disease?
- How and when did it start?
- In general, Which are the symptoms or symptom that bother you the most? (in general)
- Can you give us an example of how any of these symptoms affects your daily life?

For caregivers: So now let’s talk a little bit more about your patient’s disease and how this affects his or hers daily life.

- Can you tell us briefly a little bit more about your patient’s disease?
- How and when did it start?
- (in general)Which symptom or symptoms do you consider bother him or her the most? (in general)
- Can you give us an example of how any of these symptoms affect his or her daily life?
  1. At this point, participants will be introduced to a list of attributes related to potential benefits and risks from a hypothetical treatment. **(FGD slide 3)**

Here you have a list of “attributes” that may be relevant when making a decision about a potential treatment for a neuromuscular disorder like those represented in this group. These “attributes” may represent possible benefits (or good effects) or potential risks (or bad effects) from a potential treatment for your disease. We would like you to go through this list in silence for a few minutes. Consider as well if there are any additional benefits (or good effects) or risks (bad effects) that you consider relevant but are missing on this list and feel free to write them down on the spaces provided. You can also make notes on the additional blank pages if needed as these may help for the next discussion. If you do not understand a term, please ask.

Give the audience between 2 to 5 minutes to read the list through.

Now, using the ranking spaces on the side, we would like you to individually select from this list what you think are the five most important attributes for you (for your patient) and put them in rank order with one (1) being the attribute you personally consider to be most important to you and five (5) the least important of those.

Give the audience between 5 to 10 minutes to complete the list revision.

- 1. Expected benefits (or good effects):

We will now talk about these “attributes” and will give you the opportunity to comment on these a bit more.

For patients: Imagine a new treatment for your disease could be available tomorrow:

- Which symptom would you like to be cured first?
- If the treatment could not cure symptoms fully, what disease symptom(s) would you most want at least to improve?
- And, with what type of improvement you would be satisfied? For example, happening less often or with less intensity.

For caregivers: Imagine a new treatment for his or her disease could be available tomorrow:

- Which symptom do you think he or her would like to cure first?
- If the treatment could not cure symptoms fully, what disease symptom(s) do you think he or her would want at least to improve?
- And, with what type of improvement he or she would be satisfied? For example, happening less often or with less intensity.
  1. Risk (or bad effects) tolerance:

For patients: Now, imagine a new treatment for your disease could be available tomorrow but some possible associated risks (or bad effects) could be: 1) headaches or pain in the muscle or bone; 2) stomach or gut effects such as nausea, lack of appetite and diarrhoea; 3) heart-related effects such as: chest pain or palpitations; 4) emotional or behavioural effects such as: insomnia, anxiety, depression and irritability; 5) or damage to the liver, kidneys or eyes.

- How do you feel about these? Is there any of these that you fear the most?
- Have you ever experienced any of these symptoms as a secondary effect of a medication?
  - If yes, how bad this (or each of these) was (or were)?
  - *If yes, how often this (or each of these) happen?*
  - *If you have to score it from 0 to 10, from 0 being not bad at all and 10 being the worse possible sensation ever*, how would you score it?
- Now, if there could be a treatment attempting to improve the worst of your disease symptom (the symptom you wrote down before), is there any associated risk(s) you would not tolerate?
- Can you think of any additional risks (or bad effects) not mentioned yet that you would not tolerate no matter what benefit you receive from the treatment?

For caregivers: Now, imagine a new treatment for his or her disease could be available tomorrow but some possible associated risks (or bad effects) could be: 1) headaches or pain in the muscle or bone; 2) stomach or gut effects such as nausea, lack of appetite and diarrhoea; 3) heart-related effects such as: chest pain or palpitations; 4) emotional or behavioural effects such as: insomnia, anxiety, depression and irritability; 5) or damage to the liver, kidneys or eyes.

- How do think he or she would feel about these? Is there any of these that you think he or she would fear the most?
- Has he or she ever experienced any of these symptoms as a secondary effect of a medication?
  - If yes, how bad this (or each of these) was (or were)?
  - *If yes, how often this (or each of these) happen?*
  - *if he or she would have to score it from 1 to 5, 5 being the worse possible sensation ever and, 1 being not bad at all*, how do you think he or she would have scored it?
- Now, if there could be a treatment attempting to improve his or her worst disease symptom (referring to the symptom you wrote down before), is there any associated risk(s) you think he or she would not tolerate?
- Can you think of any additional adverse event not mentioned yet that he or she would not tolerate no matter what benefit received from the treatment?
  1. Time for a Break (approx. 15 minutes)
  2. Ranking attributes exercise [appendix I] **(FGD slide 4)**

Welcome back!

Now that we have a clearer and wider idea of possible benefits (or good effects) and potential risks (or bad effects) relevant for you when taking a decision for a disease treatment, we would like you to go back to the list revised at the start of the discussion and silently and independently rank them again from one (1) to five (5) the items on the list depending on the level of importance for you in specific. With one (1) meaning the most important and five (5) the least of those five. Remember, you can add items to the list that we think relevant but that we missed.

- 1. Round up questions **(FGD slide 5)**

To summarise, today was about getting a better understanding of your (your patient’s) current disease needs and the priorities that you (your patient) would like to either cure or at least improve your (his/her) disease. In addition, we learned about potential bad effects from a treatment that you (your patient) would not be able to tolerate.

These were all the questions I had for you, but before we finish:

- Do you have anything you want to **add** or **emphasise**?
- Do you have any **questions for us**?
- Would you feel comfortable being contacted again if we have any **follow up questions**?

Thank you for your participation. If you have any other questions, comments, or want to get in touch with me, I will give you my contact details.

If you are interested in helping us test the survey we will use in the next trial, you can complete this form with your name and email address and we will get back to you once we are ready to start the pilot. This is again entirely voluntary and you will not be obliged to take part, even if you provide us with an email address today.

- 1. [Give a break before continuing for this] If participant wishes to continue, one or two of the elicitation preference-tool examples will be presented and their initial appreciation will be explored (and recorded).

**If discussion gets stuck, use probes to elicit expansion:**

| Continuers | *“Go on,” “I hear you,” “Hmmm,” “Aha”* |
| --- | --- |
| Legitimation | *“That makes sense.”* |
| Open-ended questions | *“Tell me more about...”* |
| Understanding | *“It seems like …” “What did you think then?” “You mentioned ___, can you described for me what you mean with that?” “When you said ___. How did you mean?”* |
| Exploration | *“I wonder if you …”; “Can you give me an example?”* |
| Rephrasing | *“Let me summarize what you have told me so far…”* |
| Checking the patient's understanding | *“Could you summarize what we have discussed so far?”* |
