# Supplementary material for: Patient Preferences in Rare Diseases: A Qualitative Study in Neuromuscular Disorders to Inform a Quantitative Preference Study
Source: Patient. 2021 Feb 27;14(5):601–12. doi: 10.1007/s40271-020-00482-z (PMC8357717; doi:10.1007/s40271-020-00482-z)
Supplement: Supplementary file 2 — Supplementary file2 (DOCX 125 kb) [file 40271_2020_482_MOESM2_ESM.docx]

Supplementary Material 2. Transcripts details

| **Code** | **Disease sign or symptom** | **Daily life activities interference** |
| --- | --- | --- |
| Muscle Strength (N=69; mb=12)  With DLA example N=52  Impact on Work N=6  Impact on social and leisure  N=19 | “the weakness in my hands”  “my arms feel weaker”  “the weakness because I used to be so strong”  “I was getting weaker and weaker”  “I couldn’t lift my feet…I have…foot drop?”  “and his strength on his hands…”  “because he is quite weak” | “It's the things that I can't do that bothers me… [an example?] like peeling potatoes.. I would say…. I don't cut stuff like that cause I got no pressure (on hands)…”  “I mean I still can lift stuff like drinks and stuff like that [and demonstrates how he can lift a glass], its lifting up.. something like that [points something] … then, I wouldn’t”  “To have to ask a stranger on the tube to open a bottle of water for me..”  “but for the last couple of years, it has gotten worse … I fell over, cause [uhm] I couldn’t lift my feet.”  “he can’t walk unless he is supported on his waist”  “he can walk with his 2 sticks around, our bungalow back and forwards to the toilet… for any long distances he needs to scooter, because he's quite weak.” |
| Energy, Endurance and Daytime Sleepiness  (N=56, mb=4)  With DLA examples N=43  Impact on Work N=5  Impact on social and leisure  N=9 | “basically just tired”  “it was just the tiredness that got me realized that it was not normal”  “the energy level just dropped”  “I am sleepy all the time”  “he falls asleep just like that”  “I was tired for no reason” | “I can’t keep up with my children… nor the dog”  “I knew something was wrong, I just couldn’t keep up with my friends…”  “when I am sleepy it affects my physical abilities”  “If I had a busy day it will take me awhile to recover”  “he could sleep forever… I would struggle to wake him up”  “with tiredness I always used to put it down to having kids or working shifts… but there were still little alarm bells ringing” |
| Cognition and learning difficulties  (N=23, mb=4)  With DLA examples N=18  Impact on Work N=11  Impact on social and leisure  N=11 | “my main muscle that really scares me these days, is my frontal cortex muscle”  “because of mental changes”  “I can’t think quickly”  “I mean his memory…”  “I am not very brainy actually”  “concentration is not there”  “and his inability to organize himself”  “he is quite cognitively affected” | “I am losing my memories so fast”  “will not stop talking, you might have noticed. And he will repeat himself one subject after another after another”  “for me the main issue is kind of checking him out mentally and. just getting him to respond to you and he's delayed...”  “I could be sitting in the meeting and suddenly clicks in and that's it[!] and everybody could be talking Chinese for all I know ...consequently its really impacting on my work, you know.”  “my wife says I am loosing that (my cognitive capabilities). She is concerned so it worries me that she has mentioned it…”  “He managed to get to university but took him longer than expected…”  “he was a very slow learner”  “I was called by his tutors, saying ‘we're not quite sure about [name], we know he's got myotonic dystrophy but we don't really know why he's not coming to lectures.' that's when I begin to realize, he got some organizational problems.” |
| Balance and coordination  (N=21, mb=1)  With DLA examples N=18  Impact on Work N=5  Impact on social and leisure  N=15 | “I have fell over”  “I am in average falling every ten days”  “the main thing for me is balance”  “the difficulties in balance” | “I got a fear of falling, because it's so humiliating…and I’ve done it (fell over) plenty of times but its the humiliation that's awful”  “his balance is very off he couldn't walk up a ladder anymore ...and he can't play golf… he can't stand and hit a ball”  “he was a builder and he fell 3 stories” |
| Cardiovascular fitness  (N=21, mb=0)  With DLA examples N=4  Impact on Work N=0  Impact on social and leisure  N=3 | “I have heart problems as well”  “there are aspects with cardio and vascular”  “I am obviously concerned about my heart”  “I have a pacemaker” | “his blood pressure changes with heights quite drastically like in altitudes when going skiing”  “I might also have a pacemaker at some point soon” |
| Speech  (N=18, mb=3)  With DLA examples N=18  Impact on Work N=0  Impact on social and leisure  N=17 | “speech has always been a problem”  “speech as well… it tend to get slurred”  “I cannot speak properly”  “suffers with dysarthria” | “his speech is indistinct and when he gets excited you...you can't follow and I would have to say: “shut up and start again”…”  “… not being able to talk...she does some basic signing and she can attempt to say a few words as well...but it takes her quite a long time”  “when I’m talking to somebody, especially on the phone, I have to repeat myself, um friends don't really understand me” |
| Gut  (N=18, mb=3)  With DLA examples N=13  Impact on Work N=0  Impact on social and leisure  N=3 | “and my bowel has been my biggest problem”  “toilet problems”  “what affects me the most is my stomach issues”  “gut” | “Stomach problem is holding me as well. I’ve been sick with it all the time.”  “I got diarrhea and constipation from 1 day to the other”  “well… he still uses nappies…”  “if I am going out, I have to think ahead and the first word that gets into your head is “toilet”. Cause I go to specific shops because of the loo, because I know that if I need to go I can't wait”  “lots of stomach doctors and always said it was IBS” |
| Mood and Motivation^  (N=15, mb=3)  With DLA examples N=8  Impact on Work N=0  Impact on social and leisure  N=1 | “I have apathy”  “depression”  “they are not as driven as you would like them to be”  “it was the lack of motivation” | “they don't need help with personal care, but the motivational thing is a constant needing to keep them going [others: yeah, yeah…]”  “I can not get motivated to do anything”  “I don’t want to do anything, sometimes I can't bothered to do stuff, and [um], it’s just, it’s just so… depressing, you know” |
| Swallowing  (N=14, mb=1)  With DLA examples N=8  Impact on Work N=0  Impact on social and leisure  N=4 | “I have been diagnosed with a problem of swallowing”  “he is PEG fed”  “gagging when eating”  “had to stop to swallow saliva” | “I’m coughing food and having difficulty swallowing, and the word PEG to me which I kept thinking on about, and …. it’s that kind of loss of independence what sort of affect me the most.”  “my husband.. stops in his conversation. and consciously swallow. cause its.. his saliva builds up”  “I know it may sound horrible, but, his enjoyment was food and I mean… you know how much we love having a nice meal. He doesn't have that now.” |
| Myotonia^  (N=14, mb=0)  With DLA examples N=11  Impact on Work N=0  Impact on social and leisure  N=3 | “is this myotonia…”  “the stiffness in my hands and legs”  “a tightness in my muscles”  “it is difficult to let the hand go” | “To have to ask a stranger on the tube, to open a bottle of water for me”  “one day I came back from the gym, and it was really cold, and I couldn’t turn the key on my door...and I thought: ‘what's happening, what's happening with my hand?’”  “um… opening jars and opening cans…, I mean can't”  “then shaking hands and that sort of things you know can be a problem” |
| Respiratory system  (N=12, mb=0)  With DLA examples N=5  Impact on Work N=0  Impact on social and leisure  N=1 | “risk of respiratory infections”  “he would be sick in an alarming rate”  “respiratory failure”  “sleep apnea” | “My need to wear a mask, I really don't like that”  “He is on BiPap over night”  “We were told to take her out of nursery to avoid respiratory infections” |
| N= number of times the code was mentioned  mb= number of times the code was highlighted as the sign or symptom that bothered the patient the most  ^codes exclusively identified in the DM1 group; such as organ failure and hearing were exclusive from MD group. | | |
