# Supplementary material for: Patient Preferences in Rare Diseases: A Qualitative Study in Neuromuscular Disorders to Inform a Quantitative Preference Study
Source: Patient. 2021 Feb 27;14(5):601–12. doi: 10.1007/s40271-020-00482-z (PMC8357717; doi:10.1007/s40271-020-00482-z)
Supplement: Supplementary file 3 — Supplementary file3 (DOCX 65 kb) [file 40271_2020_482_MOESM3_ESM.docx]

Supplementary Material 3. Expected Benefits Ranking Exercise

Please rank them from one (1) to five (5) with one **(1)** being the **most** important to you and five **(5)** the **least** important.

______ Reduce pain

______ Improve muscular strength

______ Improve energy and endurance

______ Reduce tiredness

______ Reduce daytime sleepiness

______ Reduce muscle wasting

______ Improve cardiac function

______ Improve respiratory function

______ Improve gut function

______ Improve cognition

______ Slow down disease progression

______ Improve balance

______ Improve speech performance

______ Improve vision

______ Improve swallowing

other (please specify):

_______________________________________________________________________________

_______________________________________________________________________________

_______________________________________________________________________________
