# Supplementary material for: Patient Preferences in Rare Diseases: A Qualitative Study in Neuromuscular Disorders to Inform a Quantitative Preference Study
Source: Patient. 2021 Feb 27;14(5):601–12. doi: 10.1007/s40271-020-00482-z (PMC8357717; doi:10.1007/s40271-020-00482-z)
Supplement: Supplementary file 4 — Supplementary file4 (DOCX 100 kb) [file 40271_2020_482_MOESM4_ESM.docx]

Supplementary Material 4. Analysis Methods

**The predefined codes were (coding tree):**

- - - Reasons for participating
    - Unmet health priorities
      - Disease experience: symptoms or signs
      - Disease experience: daily life activity interference
      - Most bothersome symptoms
    - Expected benefits
      - Symptoms to be cured first
      - Symptoms to be improved
      - Satisfactory level of improvement
    - Risk tolerance
      - Risk perceptions (feelings)
      - Feared risks
      - Experience with risks
      - Non-tolerated risks

We used the Framework Method as described by Gale et al. (Gale NK, Heath G, Cameron E, Rashid S, Redwood S. Using the framework method for the analysis of qualitative data in multi-disciplinary health research. BMC Medical Research Methodology 2013 13:117).

*Stage 1: Transcription*

All interviews were transcribed by the person who conducted or assisted in the interview/focus group and double checked by a second researcher for quality verification.

*Stage 2: Familiarization with the interview*

Interviews and focus groups were analysed by 4 researchers (CJM, EvO, IH and JO). One informative focus groups was selected by CJM and to be analysed separately by the three researchers.

Initially the transcripts will be used for familiarization. In this stage, the right margins of the transcripts were used to write down analytical notes, thoughts or impressions (e.g. when interviewees expressed exceptionally strong or contrasting views to other interviewees). No feedback will be given towards interviewees about these notes.

*Stage 3: Coding*

We combined approach to analysis: themes will be **both identified inductively** from the accounts (experiences and views) of research participants **and deductively** from specific pre-defined sets of interests to the project and thus the research questions and questions in the interview guide. We used the pre-defined coding list and hierarchy (i.e. coding tree) presented before.

To ensure important aspects of the data will not be missed, CJM, EvO, IH and JO performed their **open-coding** independently. Then, transcripts will be printed out and coded independently: meaning that each researcher will check if all themes are covered and if not, new codes will be assigned.

Gale et al. argue that in this stage, it is valuable to involve other stakeholders to give alternative viewpoints, e.g. clinicians. In our case we involve three researchers with experience in patient preferences, one health care practitioner experienced in these diseases and one caregiver representative.

*Stage 4:  Developing a working analytical framework*

After CMJ, EvO, IH and JO have each coded the same transcripts per subgroup, they met to discuss the labels they have assigned to each passage. They discussed each coded section of each of the transcripts in terms of why they have coded it and why they perceived it as meaningful to answer the research questions. After discussion, they agreed on criteria to define these codes, and missing codes if any, each with a brief definition.

Following consensus, CMJ and EvO divided all transcripts and code them using the established analytical framework. If new codes arise, these will be discussed, resulting in the process of refining, applying, and refining the analytical framework until no new codes are generated.

*Stage 5: Applying the analytical framework*

We used NVivo for this stage: the final analytical framework (coding tree) will be uploaded in NVivo. CMJ systematically went through each transcript and highlight passages of text, selecting and attaching an appropriate code from the final analytical framework (coding). EvO revised them afterwards to verify agreement.

*Stage 6: Charting the data into the framework matrix*

After coding all transcripts, sections were subdivided between themes and subthemes following the hierarchical order specified.

In excel, this were also sorted in columns and coded on the side to facilitate counting using pivot tables and stratification of data.

*Stage 7: Interpreting the data*

CMJ and EvO interpreted the data by reviewing the matrix and making connections within and between participant and categories. This process was influenced by the research questions and by new codes generated inductively from the data. During the interpretation stage, we tried to go beyond descriptions of individual cases. Team meetings with the NMD core team including patient and caregiver representatives helped with the final interpretation of data.
